# Synthesis and Catalytic Properties of Modified Electrodes by Pulsed Electrodeposition of Pt/PANI Nanocomposite

**DOI:** 10.3390/ma12050723

**Published:** 2019-03-01

**Authors:** Ali Ourari, Ridha Zerdoumi, Ramiro Ruiz-Rosas, Emilia Morallon

**Affiliations:** 1 Laboratory of Electrochemistry, Molecular Engineering and Red-Ox Catalysis (LEMIRC), Faculty of Technology, University Ferhat ABBAS Setif-1, 19000 Setif, Algeria; zerdoumiridha@yahoo.fr; 2 Instituto Universitario de Materiales, Universidad de Alicante, Ap. 99, 03690 Alicante, Spain; ramiro@ua.es

**Keywords:** Potentiostatic pulsed electrodeposition, potentiostatic pulsed electropolymerization, platinum nanoparticles, polyaniline nanofibers, electrocatalysis, methanol oxidation

## Abstract

In this study, the modification of glassy carbon electrodes by potentiostatic pulsed deposition of platinum nanoparticles and potentiostatic pulsed polymerization of polyaniline nanofibers was investigated. During the preparation of the nano-composite materials, the control of the potentiostatic pulsed deposition and potentiostatic pulsed polymerization parameters, such as pulse potential, pulse width time, duty cycle, and platinum precursor concentration allowed the optimization of the size, shape, and distribution of the deposited Pt nanoparticles. It is noteworthy that the polymerization method, cyclic voltammetry method, or potentiostatic pulsed polymerization method show an important effect in the morphology of the deposited polyaniline (PANI) film. The obtained modified electrodes, with highly uniform and well dispersed platinum nanoparticles, exhibit good electrocatalytic properties towards methanol oxidation.

## 1. Introduction

A fuel cell is an electrochemical energy conversion device that converts chemical energy into useful electrical energy via catalytic reactions. It is considered to be a key enabling technology for sustainable and reliable power generation in the twenty-first century [1,2]. Catalysis plays a crucial role in electrochemical energy conversion. It can significantly reduce the environmental impact of chemical processes by reducing the energy required to carry them out [3,4,5].

Platinum-group metals are known as catalysts in many electrochemical reactions. In spite of their efficiency and selectivity, they suffer from multiple disadvantages such as scarcity and high cost, limiting their large-scale applications [6,7]. Their use in nanoparticulate form increases the atomic efficiency. At the nano-scale level, the surface area to volume ratio increases, which consequently improves the catalytic performance. This nano-size effect leads to the more efficient use of the electrocatalyst in order to make the maximum of precious noble-metal atoms count [8,9,10,11,12,13,14,15,16,17].

Another way to enhance the catalytic performance of these electrocatalysts is to incorporate the metallic nanoparticles into conducting polymer films to avoid agglomeration. Those polymers exhibit a high surface area that leads to highly dispersed deposits [18,19,20,21]. Typical π-conductor polymers include polyaniline (PANI), polyacetylene, polypyrrole, polythiophene, etc. Among these, PANI occupies a particular place due to its facile synthesis, environmental stability, high electrochemical activity, adjustable conductivity, and its use in aqueous media [22,23].

PANI can be elaborated via chemical and electrochemical methods. The latter set of methods has remarkable advantages, since it is a simple preparation procedure where easy control of the initiation and termination polymerization steps is achievable. PANI could be electrochemically prepared via constant potential (potentiostatic) [24,25] or constant current (galvanostatic) methods [26], cyclic voltammetry [27,28,29,30], pulsed potential (pulse potentiostatic) [31,32], and pulsed current (pulse galvanostatic) [33,34] techniques. Among these methods, it was revealed that pulsed electropolymerization is particularly more reliable to grow PANI films with interesting morphologies such as nanowires and nanofibers [32].

Dispersed noble metal nanoparticles can be prepared by chemical and electrochemical methods [16,35,36,37,38]. Again, electrochemical methods present several advantages, because they do not require additives and stabilizers. Instead, these additives could be replaced by a simple controlled addition of electrons. Therefore, high-purity deposits could be achieved with low implementation costs [39,40]. Various electrochemical deposition methods have been successfully used to prepare dispersed nanoparticles using constant potentiostatic [41,42,43,44], and galvanostatic electrodeposition [45,46,47], pulsed electrodeposition [23,48,49,50], cyclic voltammetry [51], and square wave potential deposition [52,53].

In this paper, we report the electrocatalytic performance of glassy carbon (GC) modified electrodes elaborated via potentiostatic pulsed electrodeposition of platinum nanoparticles within a porous PANI film grown using two different methods (cyclic voltammetry and pulsed electropolymerization. The control of the electrodeposition parameters such as the pulse potential, pulse width, duty cycle, and platinum salt concentration allowed the optimization of the deposition conditions for better electrocatalytic performance in the electrochemical oxidation of methanol.

## 2. Experimental

### 2.1. Chemicals

All solutions were prepared dissolving analytical grade reagents in ultrapure water obtained from a water purification system (Millipore, 18.2 MΩ cm, Veolia Water Technologies, Getxo, Spain). The used chemicals are potassium hexachloroplatinate (IV) (Aldrich. Chem. Co, 98%, Taufkirchen, Germany), sulphuric acid (Carlo Erba reagents SpA, 96%, Barcelona, Spain), Aniline (Prolabo, 98.5%, Sion, Switzerland), and methanol (Aldrich. Chem. Co, 99.9%, Taufkirchen, Germany).

### 2.2. Electrode Pre-Treatment

Prior to modification, the GC electrodes (glassy carbon discs of 0.3 cm in diameter inserted in Teflon holders) were firstly treated for 15 min with “piranha solution” (a mixture of highly concentrated hydrogen peroxide and sulphuric acid). Following this, a highly concentrated nitric acid solution was used (for 15 min) to eliminate the pre-deposited remaining particles and/or any undesirable inorganic traces. After that, they were treated with sandpaper of different particle sizes. Next, the electrodes were polished with alumina powder (particle sizes 1 µm and 0.3 µm, respectively) to obtain a mirror smooth finish. Finally, they were sonicated in an ultrasonic bath with ultrapure water for 5 min.

### 2.3. Electrode Modification

The GC/PANI electrodes were obtained by scanning the glassy carbon electrodes in a 0.1 M aniline + 0.5M H_2_SO_4_ solution with a scan rate of 50 mV/s using cyclic voltammetry from −200 to 1100 mV for the first activation cycle and from −200 to 900 mV for the ten subsequent cycles. These electrodes were named as GC/PANI(CV).

**Figure 1:**
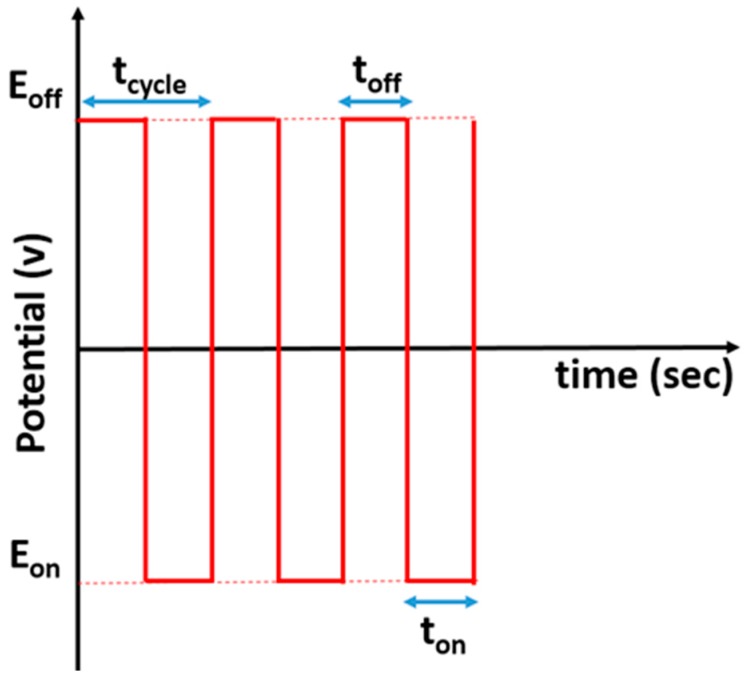
Schematic description of potential pulsed deposition method.

The effect of the polymerization method of aniline was also studied using a pulse method. The potentiostatic pulsed polymerization conditions were studied and optimized for achieving a PANI film with the higher surface area (i.e., higher double layer and redox processes as measured by cyclic voltammetry in 0.5 M H_2_SO_4_). The optimized polymerization conditions were the following (Figure 1): E_on_ = +800 mV, E_off_ = −200 mV, t_on_ = 1 s, aniline 0.1 M in 0.5 M H_2_SO_4_, duty cycle (DC) = 50% (toff = 1 s) and the polymerization time = 25 s. This electrode was named as GC/PANI(PPP).

After polymerization of PANI, the electrodes were studied in 0.5M H_2_SO_4_ solution free of aniline for several cycles. Next, platinum nanoparticles were electrodeposited on the GC/PANI electrodes with potential pulsed deposition methods. The PtNPs in the name of the electrodes indicates that the electrodes containing electrodeposited platinum nanoparticles. Figure 1 shows a schematic illustration of a potential pulse deposition profile where E_on_ and E_off_ are the lower and the upper deposition potentials, respectively, t_on_ and t_off_ are the time during which the E_on_ and E_off_ were applied. The cycle duration t_cycle_ could be determined by: t_cycle_ = t_on_ + t_off_, while the total deposition time is: t_dep_ = n t_on_, where n is the number of the deposition cycles. The DC(%) could be calculated as follows:

(1) $$DC(\%)=\frac{t_{on}}{t_{on}+t_{off}}\cdot 100$$

### 2.4. Electrochemical Characterization

Electrochemical characterization was performed using a conventional three-electrode electrochemical cell comprising a pre-treated/modified GC electrode as the working electrode, a platinum wire as a counter electrode, and an aqueous saturated calomel electrode (SCE) as a reference electrode. The system was computer-controlled by a Potentiostat/Galvanostat (Autolab PGSTAT302, Eco Chemie, The Netherlands), using Nova 2.0 software. All potentials are referred to the SCE (0.224V vs NHE). High purity nitrogen gas was purged in all solutions to assure an inert atmosphere during electrochemical experiments. All experiments were performed at room temperature without stirring the solutions.

### 2.5. Characterization of Morphology and Amount of the Deposited Platinum Nanoparticles

Field Emission Scanning Electron Microscopy (FE-SEM) (Zeiss MERLIN VP Compact, Jena, Germany), and Transmission Electron Microscopy (TEM) (Jeol JEM-1400 plus, Akishima, Japan) were used to investigate the morphology and particle size of the electrodeposited platinum nanoparticles. The amount of the deposited Pt nanoparticles was determined by inductively coupled plasma-optical emission spectroscopy (ICP-OES, Perkin Elmer Optima 4300 system, Hopkinton, MA, USA). The substrates were dissolved in 2 mL of concentrated aqua-regia and filtered. The solutions were adjusted to a final Pt concentration in the range of 10 ppm for its determination in the linear signal range.

## 3. Results and Discussion

### 3.1. Synthesis of Polyaniline by Cyclic Voltammetry and Platinum Nanoparticles by Potentiostatic Pulsed Electrodeposition Method

The effect of the PANI film on the enhancement of the catalytic performance of deposited platinum nanoparticles was investigated by comparing modified electrodes containing platinum nanoparticles deposited with the same potentiostatic pulsed electrodeposition procedure on a bare GC electrode and on a GC electrode coated with PANI film. Positive potentials of 0.9 and 1.1 V were analyzed for the synthesis of the PANI film by cyclic voltammetry. During the polymerization of aniline on the GC electrode, the first voltammogram recorded showed a well-defined peak corresponding to the oxidation of aniline at approximately 0.8 V (Figure S1) that disappears in subsequent cycles once the cation radicals have been activated in the first cycle to initiate the oligomeric formation. The synthesis performed at 1.1 V rendered a tilted voltammogram owing to overoxidation of the PANI film, while the synthesis at 0.9 V showed controlled growth of the PANI film and reversible redox processes (Figure S2). In consequence, the PANI coating of the GC electrodes was performed using a positive potential of 0.9 V. The cyclic voltammogram response of the bare GC electrode in the 5 mM K_2_PtCl_6_ + 0.5 M H_2_SO_4_ solution has been also measured (Figure S3), revealing the presence of reduction peak at −0.22 V followed by a large irreversible peak. The former peak was connected to the reduction of platinum, while the latter one came from the hydrogen evolution reaction. The presence of platinum deposited on the surface of the bare GC was confirmed during the second voltammetric cycle, where the well-known electroactive features of platinum can be observed. Given the competition between the hydrogen evolution reaction and the platinum reduction reactions observed in the voltammograms, the use of a pulse deposition procedure could be desirable in order to avoid the problems derived from the formation of gases and to increase the deposition yield. Following this, the platinum nanoparticles were electrodeposited from a 5 mM K_2_PtCl_6_ + 0.5 M H_2_SO_4_ solution on both the bare GC and the GC/PANI(CV) substrate previously synthesized. The pulse deposition conditions were E_on_ = −1 V and –750 mV, E_off_= +1 V, t_on_= 10 ms and DC= 50%. The current vs time response of a typical pulse electrodeposition experiment is shown in Figure S4. At the beginning of the experiment, the current registered during the lower potential step was larger than in the upper potential step due to the irreversible reduction current associated to the platinum electrodeposition. After application of 20% of the pulses, the current on both steps reached stable values, with the current of the lower potential step being slightly larger. As the amount of deposited platinum grew, the electroactive surface area also increased, and consequently larger currents were needed for both pulse potential steps towards the end of the experiment, Figure S4.

**Figure 2:**
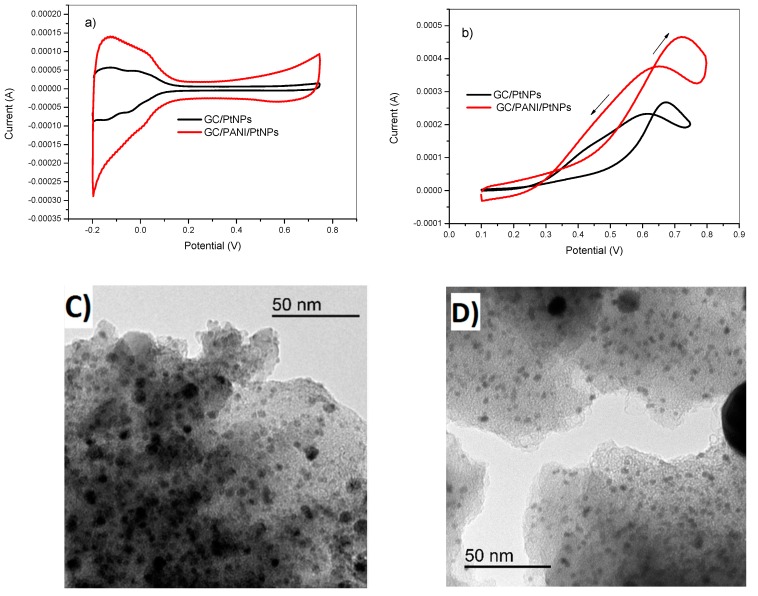
(**a**) Cyclic voltammetry in 0.5M H_2_SO_4_ and (**b**) in in 2 M methanol of glassy carbon (GC)/polyaniline (PANI) (CV)/PtNPs (red) and GC/PtNPs electrodes (black) obtained by potentiostatic pulsed electrodeposition method. The corresponding TEM images of GC/PANI(CV)/PtNPs electrodes. Deposition conditions: (**c**) E_on_ = −1 V, (**d**) E_on_ = −750 mV. E_off_= +1 V, t_on_=10 ms, DC= 50%, t_dep_=100 s, K_2_PtCl_6_= 5 mM.

Figure 2 illustrates the electrochemical behavior of both electrodes in 0.5 M H_2_SO_4_ without (Figure 2a) and with methanol (Figure 2b), respectively. The striking difference between the red and the black curve shows the importance of PANI as a support for the platinum nanoparticles. As can be clearly seen, the electrode with PANI film showed a higher electrochemical surface area compared to the electrode without PANI. The porous structure of PANI allowed the platinum nanoparticles to exhibit a higher surface area, resulting in better catalytic performance. The higher electrocatalytic activity of the GC/PANI(CV)/PtNPs anode could also be attributed to the synergetic effect of the PANI film and the deposited platinum nanoparticles.

The morphology, metal particle size, and distribution of platinum nanoparticles deposited with potentiostatic pulsed electrodeposition procedure was investigated by TEM microscopy. Figure 2c shows the TEM image of the modified GC/PANI electrode with the following potentiostatic pulsed electrodeposition conditions: t_on_ = 5 ms, E_on_ = −1 V, E_off_ = +1 V, H_2_SO_4_ = 0.5 M, t_dep_ = 5 s, and DC = 50%. The metal particle size of a supported catalyst plays an important role in the activity and selectivity of the reaction. Smaller nanoparticles could be more efficient. On the other hand, larger nanoparticles have a lower efficiency since the total exposed metal surface area decreases compared to a catalyst with similar metal loading and smaller metal particle size. The micrograph of Figure 2c shows a homogenous distribution of platinum nanoparticles within the PANI film with an average size of about 5 nm. When the E_on_ was lower, the particle size decreased to lower values of around 3 nm (Figure 2d). These illustrations also demonstrate the efficiency of the potentiostatic pulsed electrodeposition method to design nanostructured scalable surfaces.

### 3.2. Optimization of the Pulsed Electrodeposition Parameters

During the potentiostatic pulsed electrodeposition process, the control of the operating parameters, such as the pulse potential (E_on_), the pulse width (t_on_), DC, the supporting electrolyte concentration, and the platinum precursor concentration is of great importance. These parameters can influence the size, distribution, and morphology of the deposited platinum nanoparticles, which strongly affect the electrocatalytic properties of the modified electrodes. The effect of each parameter was fully investigated by the “one-factor-at a-time method”.

#### 3.2.1. Effect of the Pulse Potential (E_on_)

In this case, the platinum nanoparticles were electrodeposited from a 5 mM K_2_PtCl_6_ in 0.5 M H_2_SO_4_ solution. The lower (deposition) potential (E_on_) was set at −150, −250, −500, −750, and −1000 mV, while the other deposition conditions were fixed at: t_on_ = 5 ms, E_off_= +750 mV, t_dep_ = 100 s, and DC = 50%. The electrochemical behavior of the previously prepared modified electrodes at different deposition potentials was tested by cyclic voltammetry for methanol oxidation reaction in 2 M methanol +0.5 M H_2_SO_4_ solution.

**Figure 3:**
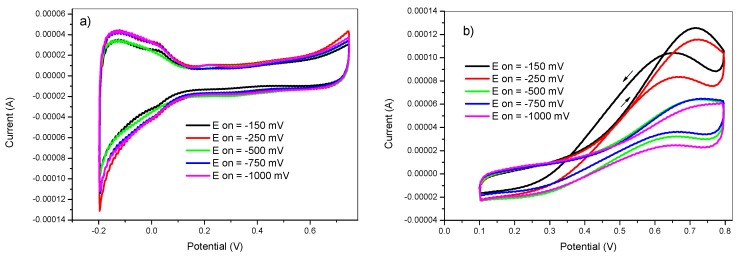
Cyclic voltammetry in (**a**) 0.5 M H_2_SO_4_ and in (**b**) 2 M methanol +0.5 M H_2_SO_4_ of GC/PANI(CV)/PtNPs electrodes obtained at various deposition potentials E_on_ = −150, −250, −500, −750 and −1000 mV.

From the obtained voltammograms in Figure 3, it is clear that the deposition potential had a strong influence on the electrochemical behavior of the prepared electrocatalysts. The oxidation peak current around 0.7 V, associated to methanol oxidation, in Figure 3b decreased as the deposition potential shifted to more negative values. The electrode prepared at −150 and −250 mV showed lower onset oxidation potential and higher oxidation current for methanol oxidation than the other electrodes prepared at different lower potential values, Figure 3b.

This behavior can be ascribed to the competition between the hydrogen evolution reaction and the Pt deposition reaction on the electrode surface. At the early deposition stages, the dominant reaction is the reduction of Pt(IV) species. As time progressed, a concentration gradient is established at the electrode/solution interface. The Pt species become depleted near the electrode surface and the hydrogen evolution reaction become more competitive. This concentration gradient is established faster at more negative potential values, favoring the growth of platinum nanoparticles instead of creating new Pt nuclei. All of these factors resulted in a relatively lower methanol oxidation current as the deposition potential was set at more negative values.

#### 3.2.2. Effect of the Upper Potential (E_off_)

**Figure 4:**
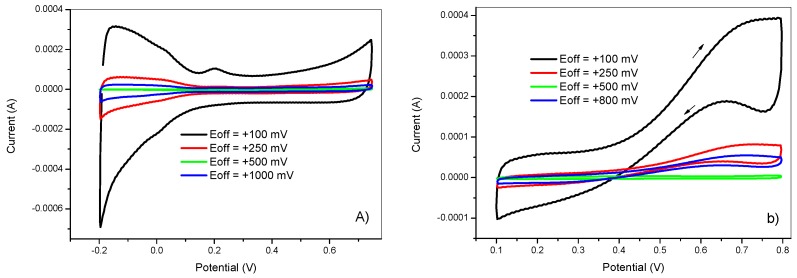
Cyclic voltammetry in (**a**) 0.5 M H_2_SO_4_ and in (**b**) 2 M methanol + 0.5 M H_2_SO_4_ of GC/PANI(CV)/PtNPs electrodes obtained at various upper potentials (E_off_) = +100, +250, +500, and +800 mV.

The effect of the upper potential was examined from the same 5 mM K_2_PtCl_6_ + 0.5 M H_2_SO_4_ solution. For each electrode, the values of the upper potentials were +100, +250, +500, +800 mV. The deposition conditions were as follows: t_on_ = 5 ms, E_on_ = −750 mV, t_dep_ = 100 s, and DC = 50%. Next, the electrochemical behavior of the obtained electrodes was tested in 0.5 M H_2_SO_4_ and in 2 M methanol +0.5 M H_2_SO_4_ solutions. Figure 4, shows the resulting voltammograms.

It is clear that the upper potential value had a great influence on the electrochemical behavior of deposited platinum nanoparticles. The electrode prepared at E_off_ = 100 mV showed the highest voltammetric charge related with the higher electrochemical surface area and methanol oxidation peak current compared to other electrodes prepared at higher E_off_ values. This could be explained by the fact that at higher upper potential values (E_off_), a concentration gradient will be formed faster than for lower values. However, low E_off_ will favor the nucleation step at the expanse of particle growth by decreasing the concentration of platinum species in the double layer to have enough supply for the next deposition half-cycle. It can also be seen that at the value of E_off_ = 500 mV, the voltammograms showed no activity for methanol oxidation reaction.

#### 3.2.3. Effect of the Pulse Width (t_on_)

**Figure 5:**
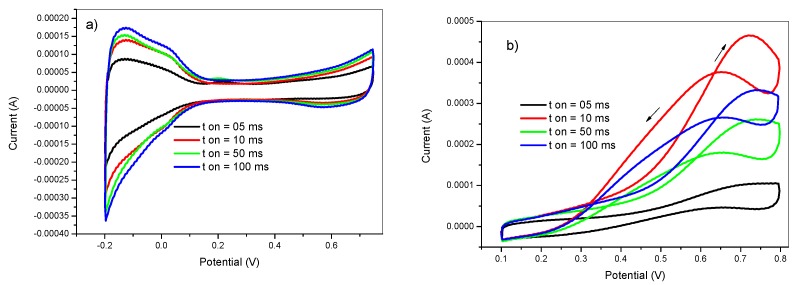
Cyclic voltammetry in (**a**) 0.5 M H_2_SO_4_ and in (**b**) 2 M methanol +0.5 M H_2_SO_4_ of GC/PANI(CV)/PtNPs electrodes obtained at various pulse widths (t_on_) = 05, 10, 50, and 100 ms.

The pulse width is the time during which the deposition potential is applied. The effect of the pulse width was investigated by performing Pt deposition at t_on_ values of 5, 10, 50, and 100 ms. The pulse deposition parameters were as follows: E_on_ = −750 mV, E_off_ = +750 mV, t_dep_ = 100 s, and DC = 50%. The obtained voltammograms of the electrodes prepared with various t_on_ values in 0.5 M H_2_SO_4_ and in 2 M methanol +0.5 M H_2_SO_4_ solutions are shown in Figure 5. At a negative applied potential (E_on_ = −750 mV), the reduction of Pt ions competed with the reduction of protons. However, the deposition of Pt was produced giving two simultaneous phenomena; the first one was the formation of new independent nuclei (nucleation process) whereas the second was the growth of the previously formed nuclei into bigger particles (growth process).

At short pulse widths, the short exposure to the reduction potential makes the nucleation more efficient than the growth. However, at very short pulse widths, the obtained nanoparticles are incorporated into the PANI film leading to lower efficiency of the prepared electrocatalysts as shown in the black voltammogram of Figure 5. On the other hand, long pulse width durations leads to the formation of larger particles by favoring the growth process. Therefore, the pulse width needs to be optimized to balance both nucleation and growth in order to achieve higher catalytic efficiency. From Figure 5, it can be noticed that the optimum value of the pulse width for maximizing the methanol oxidation reaction rate is 10 ms.

#### 3.2.4. Effect of the DC%

**Figure 6:**
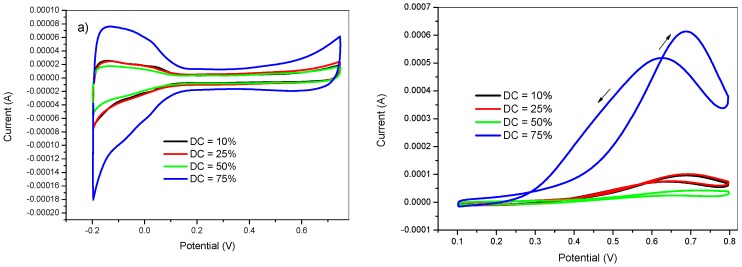
Cyclic voltammetry in (**a**) 0.5 M H_2_SO_4_ and in (**b**) 2 M methanol +0.5 M H_2_SO_4_ of GC/PANI(CV)/PtNPs electrodes obtained at various duty cycles (DC) = 10, 25, 50, and 75 %.

The effect of the DC on the electrode performance was also studied by changing the t_off_ duration for a fixed t_on_ (5 ms). The t_off_ duration was 45, 15, 5, and 1.66 ms so that, the DC values were 10, 25, 50, and 75 %, respectively. The parameters used for the experimental potentiostatic pulsed electrodeposition process were as follows: t_on_ = 5 ms, E_on_ = −750 mV, t_dep_ = 100 s, and E_off_ = +750 mV. The obtained voltammograms at various duty cycle values in 0.5 M H_2_SO_4_ and in 2 M methanol +0.5 M H_2_SO_4_ are shown in Figure 6.

The above voltammograms indicated a strong DC dependence of the voltammetric profile in absence of methanol (Figure 6a) and in methanol oxidation current (Figure 6b). The electrode prepared at a DC of 75 % had an outstanding methanol oxidation current peak and an increase in the voltammetric profile related with the Pt nanoparticles compared to other electrodes prepared at lower DC values. In this case, a low DC value corresponded to a long period of the positive potential (t_off_). At high t_off_ duration, the previously established concentration gradient during the negative half-cycle was dissipated prior the next deposition half-cycle. On the other hand, short t_off_ duration did not give enough time to the established concentration gradient to dissipate. All these parameters could play a key role in the catalytic efficiency of the obtained PtNPs.

#### 3.2.5. Effect of the K_2_PtCl_6_ Concentration

**Figure 7:**
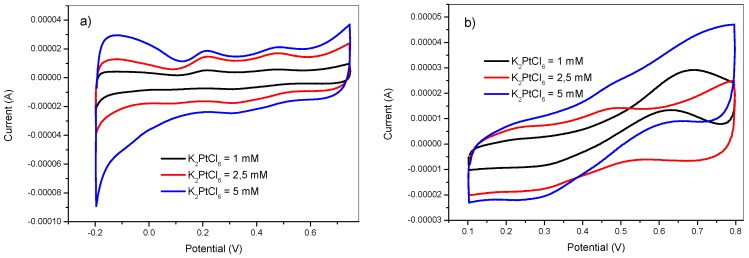
Cyclic voltammetry in (**a**) 0.5 M H_2_SO_4_ and in (**b**) 2 M methanol +0.5 M H_2_SO_4_ of GC/PANI(CV)/PtNPs electrodes obtained at various K_2_PtCl_6_ concentrations (1, 2.5, and 5 mM).

The effect of the K_2_PtCl_6_ concentration on the catalytic performance of the obtained modified electrodes was also examined using 1, 2.5 and 5 mM. The deposition conditions were as follows: t_on_ = 5 ms, E_on_ = −750 mV, E_off_ = +750 mV, H_2_SO_4_ = 0.5 M, t_dep_ = 100 s, and DC = 50%. The obtained results are illustrated in Figure 7.

Figure 7a shows that the voltammetric charge increased with K_2_PtCl_6_ concentration. At low K_2_PtCl_6_ concentrations, the depletion of the electrode surface and the formation of the diffusion boundary layer occurred in an early deposition stage with low current density compared to higher K_2_PtCl_6_ concentrations. This favored the nucleation process. On the other hand, higher K_2_PtCl_6_ concentrations ensured a permanent supply of Pt species during the cathodic half-cycle, which favored the kinetics of growth the particles. For a given electrode surface, there was an optimum concentration value of electro-active species that ensured a balance between both nucleation and particle growth during the potentiostatic pulsed electrodeposition process.

#### 3.2.6. Effect of the Deposition Time

**Figure 8:**
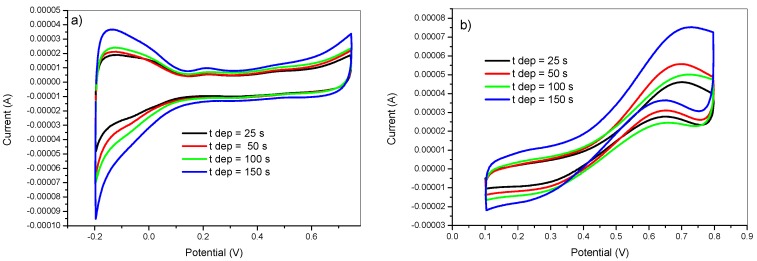
Cyclic voltammetry in 0.5 M H_2_SO_4_ (**a**) and in 2 M methanol + 0.5 M H_2_SO_4_ (**b**) of GC/PANI(CV)/PtNPs electrodes obtained at various deposition times (t_dep_) = 25, 50, 100, and 150 s.

The effect of the deposition time on the catalytic performance of the obtained modified electrodes was examined by preparing PtNPs from a 5 mM K_2_PtCl_6_ in 0.5 M H_2_SO_4_ solution at different deposition times: 25, 50, 100, 150 s. The deposition conditions were as follows: E_on_ = −750 mV, E_off_ = +750 mV, t_on_ = 5 ms, and DC = 50%. The obtained results are illustrated on Figure 8. It showed an increase in voltammetric profile associated to the adsorption–desorption of hydrogen in the Pt surface (Figure 8a) and methanol oxidation current with increasing the deposition time. This was due to the higher amount of deposited platinum for longer deposition time.

#### 3.2.7. Platinum Nanoparticles Deposited at Optimized Conditions

In order to optimize the deposition conditions for the platinum nanoparticles deposition with high catalytic performance and low platinum amount, the percentage of platinum in the synthesized modified electrodes was characterized by energy-dispersive x-ray spectroscopy (EDX) for each deposition parameter.

**Figure 9:**
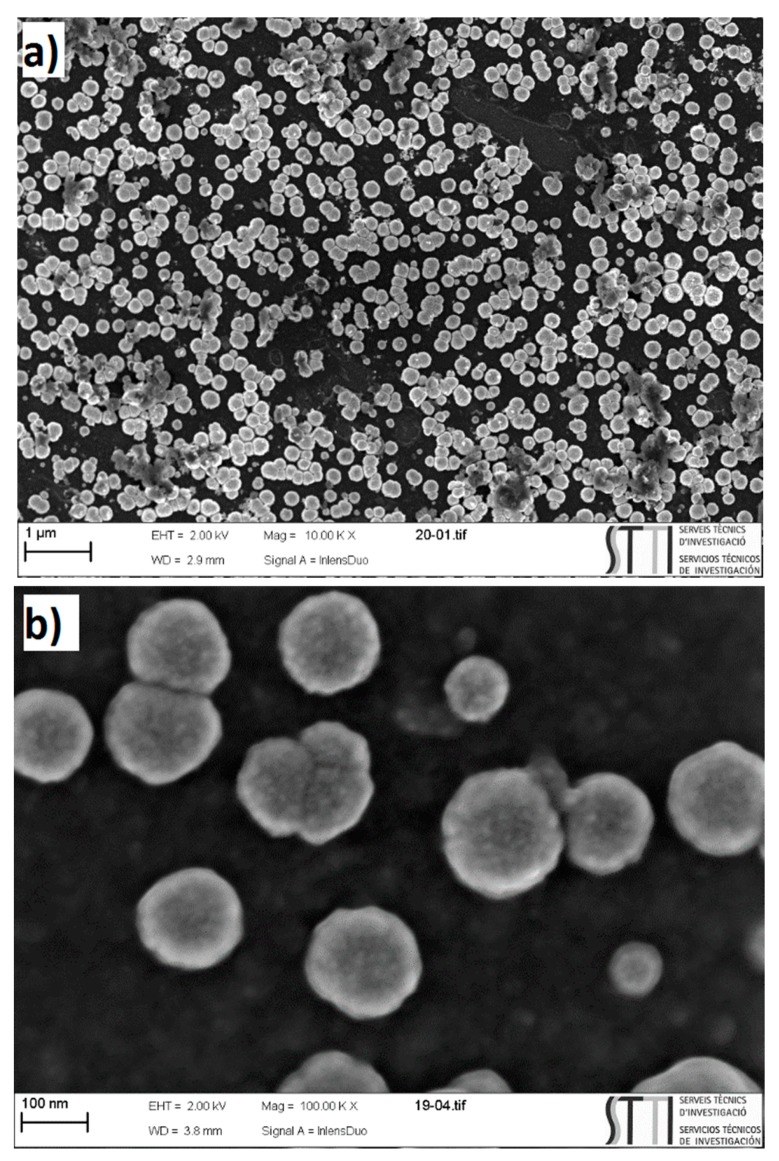
SEM micrographs of GC/PANI(CV)/PtNPs obtained with the following deposition conditions: E_on_ = −150 mV, E_off_ = +100 mV, t_on_ = 10 ms, and DC = 75%. 5 mM K_2_PtCl_6_ in 0.5 M H_2_SO_4_.

The platinum deposited in the electrodes with high ratios of methanol oxidation current and platinum percentage were the electrodes with the following deposition conditions: E_on_ = −150 mV, E_off_ = +100 mV, t_on_ = 10 ms, t_off_ = 3.33 ms, and DC = 75% 5 mM K_2_PtCl_6_ in 0.5 M H_2_SO_4_. Figure 9 shows the obtained SEM micrographs of the electrode prepared using the previous optimized conditions for a deposition time of 200 s. The bright dots in the micrographs refer to the deposited spherical platinum nanoparticles. The electrode surface exhibited a uniform platinum nanoparticle distribution all over the substrate with particle sizes around 100 nm. These particles were formed by aggregations of smaller platinum particles between 3 and 5 nm. This is in agreement with previous results [23].

**Figure 10:**
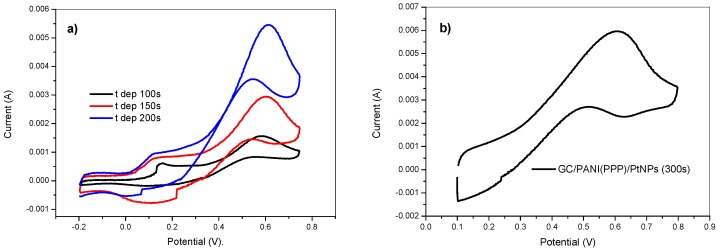
Cyclic voltammetry in 2 M methanol + 0.5 M H_2_SO_4_ (a) of the GC/PANI(CV)/PtNPs electrodes obtained at various deposition times and (**b**) of the GC/PANI(PPP)/PtNPs electrode. Deposition conditions: E_on_ = −150 mV, E_off_ = +100 mV, t_on_ = 10 ms, and DC = 75 %. 5 mM K_2_PtCl_6_ in 0.5 M H_2_SO_4_.

The catalytic efficiency was examined by scanning the electrode in a 2 M methanol +0.5 M H_2_SO_4_ solution. Figure 10a shows the obtained voltammograms at various deposition times of modified electrodes elaborated with the combination of the previous optimized deposition conditions. The voltammograms show a relatively high methanol oxidation peak at around 0.6 V (current values are compiled as I_p_ in Table 1) and a lower onset potential of the modified electrode as we increased the deposition time. The low oxidation peaks located at 0.1 V should be ascribed to the PANI film. The normalized oxidation current to the amount of deposited platinum determined using ICP-OES shows that the catalytic efficiency slightly increased with deposition time.

### 3.3. Synthesis of Polyaniline by Cyclic Voltammetry Method and by Potentiostatic Pulsed Polymerization Method: Effect in the Platinum Nanoparticles Deposition

**Figure 11:**
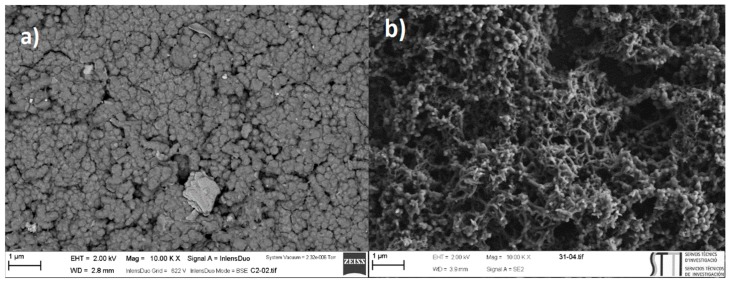
SEM micrographs of (**a**) GC/PANI(CV) electrode and (**b**) GC/PANI(PPP) electrode using 90 s of polymerization time.

In order to analyze the effect of the polymerization method, the PANI was also prepared using a potentiostatic pulse method. The potentiostatic pulsed polymerization conditions were thoroughly studied and optimized for achieving a PANI film with a higher surface area (i.e., higher double layer and redox processes as measured by cyclic voltammetry in 0.5 M H_2_SO_4_). It is worth mentioning that the applied oxidation potential during the potentiostatic pulsed polymerization process was +1000 mV only for the first initiation pulse and +800 mV for the remaining pulses. Figure 11 compares the morphology of the polyaniline obtained for both polymerization methods. The electrode prepared using cyclic voltammetry is presented in Figure 11a while Figure 11b shows the SEM micrograph of the polymer obtained using the potentiostatic pulsed polymerization method for a polymerization time of 90 s. The polymer obtained with the last method clearly shows a more porous surface than that obtained by cyclic voltammetry. Moreover, the polymer showed a fibrous morphology consisting of polyaniline nanofibers. Similar morphologies of PANI where obtained in the literature with different pulsed deposition parameters [22,31,32]. On the other hand, a less porous film made of isolated globular polymer microstructures was observed in the case of the GC/PANI(CV) electrode.

**Figure 12:**
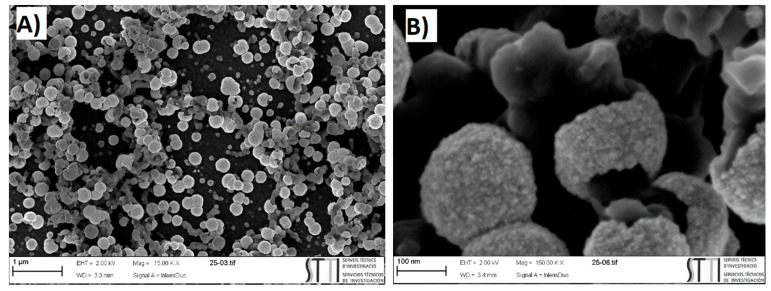
SEM micrographs of GC/PANI(PPP)/PtNPs using 25 s of polymerization time, with two magnifications: (**A**) 1 μ (a) and (**B**) 100 nm.

The electrode showing PANI nanofibers obtained using potentiostatic pulsed polymerization method with optimized conditions, for a polymerization time of 25 s, was used as support for the electrodeposition of platinum nanoparticles (GC/PANI(PPP)/PtNPs electrode). This electrode is presented in Figure 12. As can be seen, the platinum nanoparticles tended to grow all along the PANI fibers due to its relatively high conductivity in acidic medium. The platinum nanoparticles grew in a spherical shell-like morphology to surround the PANI fibers (Figure 12b) which led to a high exposed surface area compared to the platinum deposited on the polymer grown using cyclic voltammetry (Figure 9).

The advantages of the potentiostatic pulsed polymerization method upon the activity of the resulting electrode has been explored for methanol oxidation. Figure 10a shows the obtained voltammogram of the GC/PANI(CV)/PtNPs electrode in a 2 M methanol +0.5 M H_2_SO_4_ solution. It shows a relatively high oxidation peak at about 0.6 V, attributable to methanol oxidation catalyzed by platinum nanoparticles. The voltammogram during the oxidation of methanol for the electrode obtained by potentiostatic pulsed polymerization (GC/PANI(PPP)/PtNPs) is shown in Figure 10b. It can be observed that the catalytic activity was higher than the obtained using cyclic voltammetry for polymerization. The values of catalytic activity related to the amount of Pt are shown in Table 1. The ratio of the forward oxidation peak current (I_f_) to the reverse peak current (I_b_), is an index of the catalyst tolerance to the removal of poisoning species [54,55]. A higher I_f_/I_b_ ratio indicates more effective removal of poisoning species on the catalyst surface and this ratio was 2.18 for the GC/PANI(PPP)/PtNPs electrode which was higher than for the GC/PANI(CV)/PtNPs electrode (1.54), showing a better poisoning tolerance for the electrode obtained using the potentiostatic pulsed polymerization of polyaniline before the platinum deposition.

The catalytic activity results are comparable and/or slightly better than those achieved by deposition methods at constant potential [23] and somehow lower than those achieved over nanostructured carbon supports [54,55,56] and platinum deposited on PANI and on poly(o.toluidine) modified electrodes where platinum was electrodeposited at constant potential [57], even though the forward to reverse peak current ratio, was higher for the catalyst herein reported. At this early stage, these results demonstrate the feasibility of the combination of the potentiostatic pulsed deposition of platinum and potentiostatic pulsed polymerization methods, with the synthesis parameters still to be optimized in the future, including the potential use of metal alloys and nanostructured carbon supports [56,58].

**Figure 13:**
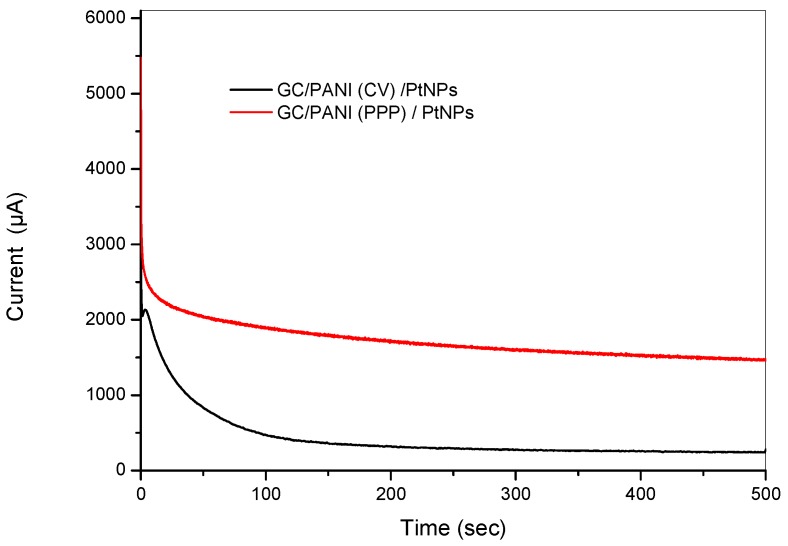
Chronoamperograms of the GC/PANI(PPP)/PtNPs (black) and of the GC/PANI(CV)/PtNPs electrode (red) and of a GC/PANI(CV)/PtNPs electrode in 2 M methanol +1 M H_2_SO_4_ at 0.6 V.

The long-term electrochemical stability of the modified electrodes has also been evaluated by chronoamperometry at 0.65 V in 2 M methanol +1 M H_2_SO_4_ solution. Figure 13 shows the obtained chronoamperograms. The initial rapid current decay might be attributed to the formation of some intermediate poisoning species during the oxidation of methanol [55]. After 500 s, the GC/PANI(PPP)/PtNPs electrode, in which the PANI was obtained by potentiostatic pulsed polymerization, showed a higher oxidation current compared to the GC/PANI(CV)/PtNPs electrode (black) in which the PANI was obtained by cyclic voltammetry, indicating a better durability towards methanol oxidation.

## 4. Conclusions

Platinum nanoparticles have been successfully electrodeposited on GC/PANI substrates by a potentiostatic pulsed electrodeposition process. The obtained results demonstrate that the studied parameters have a significant influence on the morphology, dispersion, and distribution of the deposited platinum nanoparticles, which, in return, affect the catalytic performance of the resulting modified electrodes for methanol oxidation. This study allowed us to determine optimum values of the deposition parameters which were: E_on_ = −150 mV, E_off_ = +100 mV, t_on_ = 10 ms and DC = 75%, 5 mM K_2_PtCl_6_ in 0.5 M H_2_SO_4_. Similarly, the polymerization conditions for growing PANI thin films with increased surface area were also studied. It is noteworthy that the polymerization method, cyclic voltammetry method, or potentiostatic pulsed polymerization method, showed an important effect in the morphology of the deposited PANI film. The morphology of PANI was notoriously modified, leading to the formation of nanofibers. The combination of these optimized potentiostatic pulse electrodeposition and potentiostatic pulsed polymerization conditions led to an improvement of the catalytic activity towards methanol oxidation. This high catalytic activity could be attributed to the synergistic effect of the well-dispersed platinum nanoparticles over the porous structure of the conducting polyaniline nanofibers.

## Figures and Tables

**Table 1:** The catalytic activity, amount of platinum and current oxidation peak for methanol of different electrodes.

Electrode	Pt Weight (mg)	I_p_ (mA)	Catalytic Activity (A/g)
GC/PANI (CV)/PtNPs (100 s)	0.040	1.6	39
GC/PANI (CV)/PtNPs (150 s)	0.066	2.9	45
GC/PANI (CV)/PtNPs (200 s)	0.114	5.5	48
GC/PANI (PPP)/PtNPs (300 s)	0.095	6.0	63

## Supplementary Materials

The following are available online at https://www.mdpi.com/1996-1944/12/5/723/s1, Figure S1. Cyclic voltammetry of aniline polymerisation on a GC electrode in 0.1 M aniline + 0.5M H_2_SO_4_ solution at 50 mV/s from −200 to 1100 mV for the first activation cycles. Figure S2. Cyclic voltammetry of aniline polymerization on a GC electrode in 0.1 M aniline + 0.5M H_2_SO_4_ solution at 50 mV/s from −200 to 900 mV for ten subsequent cycles. Figure S3. Cyclic voltammetry recorded on GC at 10 mV/s for 5 mM K_2_PtCl_6_ + 0.5M H_2_SO_4_ plating solution. Figure S4.Chronoamperometric curve for potentiostatically electrodeposited platinum on GC/ PANI electrodes at a pulse deposition potential (Eon) = −700mV. Deposition conditions (0.005M K_2_PtCl_6_ in H_2_SO_4_ 0.5M): t_on_ = 5 ms, E_off_ = +1V, t_dep_ = 5 s, and DC = 50%. Figure S5. SEM micrographs of GC/PANI(CV)/PtNPs obtained with the following deposition conditions: E_on_ = −500 Mv (left) E_on_ = −750 mV (right), E_off_ = +750 mV, t_on_ = 5 ms, and DC = 50%. 5 mM K_2_PtCl_6_ in 0.5M H_2_SO_4_.

## References

[1] Zhao T.S., Xu C., Chen R., Yang W.W. (2009). Mass transport phenomena in direct methanol fuel cells. Prog. Energy Combust. Sci..

[2] Peighambardoust S.J., Rowshanzamir S., Amjadi M. (2010). Review of the proton exchange membranes for fuel cell applications. Int. J. Hydrogen Energy.

[3] Debe M.K. (2012). Electrocatalyst approaches and challenges for automotive fuel cells. Nature.

[4] Tiwari J.N., Tiwari R.N., Singh G., Kim K.S. (2013). Recent progress in the development of anode and cathode catalysts for direct methanol fuel cells. Nano Energy.

[5] Braunchweig B., Hibbitts D., Neurock M., Wieckowski A. (2013). Electrocatalysis: A direct alcohol fuel cell and surface science perspective. Catal. Today.

[6] Xu Y., Zhang B. (2014). Recent advances in porous Pt-based nanostructures: synthesis and electrochemical applications. Chem. Soc. Rev..

[7] Sheng W., Zhuang Z., Gao M., Zheng J., Chen J.G., Yan Y. (2015). Correlating hydrogen oxidation and evolution activity on platinum at different pH with measured hydrogen binding energy. Nat. Commun..

[8] Merte L.R., Behafarid F., Miller D.J., Friebel D., Cho S., Mbuga F., Sokaras D., Alonso-Mori R., Weng T.-C., Nordlund D. (2012). Electrochemical Oxidation of Size-Selected Pt Nanoparticles Studied Using in Situ High-Energy-Resolution X-ray Absorption Spectroscopy. ACS Catal..

[9] Shao M., Odell J., Humbert M., Yu T., Xia Y. (2013). Electrocatalysis on Shape-Controlled Palladium Nanocrystals: Oxygen Reduction Reaction and Formic Acid Oxidation. J. Phys. Chem. C.

[10] Shao M., Peles A., Shoemaker K. (2011). Electrocatalysis on platinum nanoparticles: particle size effect on oxygen reduction reaction activity. Nano Lett..

[11] Solla-Gullón J., Vidal-Iglesias F.J., Feliu J.M. (2011). Shape dependent electrocatalysis. Annu. Rep. Sect. “C” (Phys. Chem.).

[12] Koper M.T.M. (2011). Structure sensitivity and nanoscale effects in electrocatalysis. Nanoscale.

[13] Neouze M.-A. (2013). Nanoparticle assemblies: main synthesis pathways and brief overview on some important applications. J. Mater. Sci..

[14] Kango S., Kalia S., Celli A., Njuguna J., Habibi Y., Kumar R. (2013). Surface modification of inorganic nanoparticles for development of organic–inorganic nanocomposites—A review. Prog. Polym. Sci..

[15] Cox J.T., Zhang B. (2012). Nanoelectrodes: Recent advances and new directions. Annu. Rev. Anal. Chem. (Palo Alto. Calif)..

[16] Zaera F. (2013). Nanostructured materials for applications in heterogeneous catalysis. Chem. Soc. Rev..

[17] Wu B., Zheng N. (2013). Surface and interface control of noble metal nanocrystals for catalytic and electrocatalytic applications. Nano Today.

[18] Lu X., Zhang W., Wang C., Wen T.-C., Wei Y. (2011). One-dimensional conducting polymer nanocomposites: Synthesis, properties and applications. Prog. Polym. Sci..

[19] Sarkar S., Guibal E., Quignard F., SenGupta A.K. (2012). Polymer-supported metals and metal oxide nanoparticles: synthesis, characterization, and applications. J. Nanoparticle Res..

[20] Ferreira V.C., Melato A.I., Silva A.F., Abrantes L.M. (2011). Attachment of noble metal nanoparticles to conducting polymers containing sulphur – preparation conditions for enhanced electrocatalytic activity. Electrochim. Acta.

[21] Reddy K.R., Sin B.C., Ryu K.S., Kim J.-C., Chung H., Lee Y. (2009). Conducting polymer functionalized multi-walled carbon nanotubes with noble metal nanoparticles: Synthesis, morphological characteristics and electrical properties. Synth. Met..

[22] Jiang H.-F., Liu X.-X. (2010). One-dimensional growth and electrochemical properties of polyaniline deposited by a pulse potentiostatic method. Electrochim. Acta.

[23] Domínguez-Domínguez S., Arias-Pardilla J., Berenguer-Murcia Á., Morallón E., Cazorla-Amorós D. (2008). Electrochemical deposition of platinum nanoparticles on different carbon supports and conducting polymers. J. Appl. Electrochem..

[24] López-Palacios J., Muñoz E., Heras M.A., Colina Á., Ruiz V. (2006). Study of polyaniline films degradation by thin-layer bidimensional spectroelectrochemistry. Electrochim. Acta.

[25] Weng S., Lin Z., Chen L., Zhou J. (2010). Electrochemical synthesis and optical properties of helical polyaniline nanofibers. Electrochim. Acta.

[26] Luo K., Shi N., Sun C. (2006). Thermal transition of electrochemically synthesized polyaniline. Polym. Degrad. Stab..

[27] Li M.C., Ma C.A., Liu B.Y., Jin Z.M. (2005). A novel electrolyte 1-ethylimidazolium trifluoroacetate used for electropolymerization of aniline. Electrochem. Commun..

[28] Baba A., Tian S., Stefani F., Xia C., Wang Z., Advincula R.C., Johannsmann D., Knoll W. (2004). Electropolymerization and doping/dedoping properties of polyaniline thin films as studied by electrochemical-surface plasmon spectroscopy and by the quartz crystal microbalance. J. Electroanal. Chem..

[29] Milczarek G. (2007). Electrochemical modification of poly-aniline films in the presence of guaiacol–sulfonic acid. Electrochem. Commun..

[30] Dalmolin C., Canobre S.C., Biaggio S.R., Rocha-Filho R.C., Bocchi N. (2005). Electropolymerization of polyaniline on high surface area carbon substrates. J. Electroanal. Chem..

[31] Zhou H.H., Wen J.B., Ning X.H., Fu C.P., Chen J.H., Kuang Y.F. (2007). Comparison of the growth process and electrochemical properties of polyaniline films prepared by pulse potentiostatic and potentiostatic method on titanium electrode. J. Appl. Polym. Sci..

[32] Karami H., Asadi M.G., Mansoori M. (2012). Pulse electropolymerization and the characterization of polyaniline nanofibers. Electrochim. Acta.

[33] Zhou H.H., Jiao S.Q., Chen J.H., Wei W.Z., Kuang Y.F. (2004). Effects of conductive polyaniline (PANI) preparation and platinum electrodeposition on electroactivity of methanol oxidation. J. Appl. Electrochem..

[34] Zhou H., Jiao S., Chen J., Wei W., Kuang Y. (2004). Relationship between preparation conditions, morphology and electrochemical properties of polyaniline prepared by pulse galvanostatic method (PGM). Thin Solid Films.

[35] Welch C.M., Compton R.G. (2006). The use of nanoparticles in electroanalysis: a review. Anal. Bioanal. Chem..

[36] Guo S., Wang E. (2007). Synthesis and electrochemical applications of gold nanoparticles. Anal. Chim. Acta.

[37] Coutanceau C., Brimaud S., Lamy C., Léger J.-M., Dubau L., Rousseau S., Vigier F. (2008). Review of different methods for developing nanoelectrocatalysts for the oxidation of organic compounds. Electrochim. Acta.

[38] Coutanceau C., Urchaga P., Brimaud S., Baranton S. (2012). Colloidal Syntheses of Shape- and Size-Controlled Pt Nanoparticles for Electrocatalysis. Electrocatalysis.

[39] Ji H., Li M., Wang Y., Gao F. (2012). Electrodeposition of graphene-supported PdPt nanoparticles with enhanced electrocatalytic activity. Electrochem. Commun..

[40] Zhong C., Hu W., Cheng Y. (2013). Recent advances in electrocatalysts for electro-oxidation of ammonia. J. Mater. Chem. A.

[41] Liu Z.-L., Huang R., Deng Y.-J., Chen D.-H., Huang L., Cai Y.-R., Wang Q., Chen S.-P., Sun S.-G. (2013). Catalyst of Pt nanoparticles loaded on multi-walled carbon nanotubes with high activity prepared by electrodeposition without supporting electrolyte. Electrochim. Acta.

[42] Brülle T., Stimming U. (2009). Platinum nanostructured HOPG – Preparation, characterization and reactivity. J. Electroanal. Chem..

[43] Miyake M., Ueda T., Hirato T. (2011). Potentiostatic Electrodeposition of Pt Nanoparticles on Carbon Black. J. Electrochem. Soc..

[44] Raoof J.B., Ojani R., Hosseini S.R. (2013). Electrochemical synthesis of a novel platinum nanostructure on a glassy carbon electrode, and its application to the electrooxidation of methanol. Microchim. Acta.

[45] Paoletti C., Cemmi A., Giorgi L., Giorgi R., Pilloni L., Serra E., Pasquali M. (2008). Electro-deposition on carbon black and carbon nanotubes of Pt nanostructured catalysts for methanol oxidation. J. Power Sources.

[46] Gopi D., Indira J., Kavitha L. (2012). A comparative study on the direct and pulsed current electrodeposition of hydroxyapatite coatings on surgical grade stainless steel. Surf. Coatings Technol..

[47] Sanaty-Zadeh A., Raeissi K., Saidi A. (2009). Properties of nanocrystalline iron–nickel alloys fabricated by galvano-static electrodeposition. J. Alloys Compd..

[48] Liu J., Zhong C., Du X., Wu Y., Xu P., Liu J., Hu W. (2013). Pulsed electrodeposition of Pt particles on indium tin oxide substrates and their electrocatalytic properties for methanol oxidation. Electrochim. Acta.

[49] Fouda-onana F., Guillet N., Almayouf A.M. (2014). Modi fi ed pulse electrodeposition of Pt nanocatalyst as high-performance electrode for PEMFC. J. Power Sources.

[50] Burk J.J., Buratto S.K. (2013). Electrodeposition of Pt Nanoparticle Catalysts from H 2 Pt(OH) 6 and Their Application in PEM Fuel Cells. J. Phys. Chem. C.

[51] Ding K., Jia H., Wei S., Guo Z. (2011). Electrocatalysis of Sandwich-Structured Pd/Polypyrrole/Pd Composites toward Formic Acid Oxidation. Ind. Eng. Chem. Res..

[52] Cui H.-F., Ye J.-S., Zhang W.-D., Wang J., Sheu F.-S. (2005). Electrocatalytic reduction of oxygen by a platinum nanoparticle/carbon nanotube composite electrode. J. Electroanal. Chem..

[53] Tian N., Zhou Z.-Y., Sun S.-G., Ding Y., Wang Z.L. (2007). Synthesis of tetrahexahedral platinum nanocrystals with high-index facets and high electro-oxidation activity. Science.

[54] Sevilla M., Sanchís C., Valdés-Solís T., Morallón E., Fuertes A.B. (2009). Highly dispersed platinum nanoparticles on carbon nanocoils and their electrocatalytic performance for fuel cell reactions. Electrochim. Acta.

[55] Sevilla M., Martinez-de Lecea C.S., Valdés-Solís T., Morallón E., Fuertes A.B. (2008). Solid-phase synthesis of graphitic carbon nanostructures from iron and cobalt gluconates and their utilization as electrocatalyst supports. Phys. Chem. Chem. Phys.

[56] Sevilla M., Sanchís C., Valdés-Solís T., Morallón E., Fuertes A.B. (2008). Direct synthesis of graphitic carbon nanostructures from saccharides and their use as electrocatalytic supports. Carbon.

[57] Zhang G., Tan L., Cheng H., Li F., Liu X., Lu J. (2018). Different interesting enhanced influence from polyaniline and poly (o-toluidine) on electrocatalytic activities of Pt on them toward electrooxidation of methanol. Int. J. Hydrogen Energy..

[58] Ayán-Varelaa M., Ruiz-Rosas R., Villar-Rodil S., Paredesa J.I., Cazorla-Amorós E., Morallón A., Martínez-Alonso J.M.D.T. (2017). Efficient Pt electrocatalysts supported onto flavin mononucleotide–exfoliated pristine graphene for the methanol oxidation reaction. Electrochim. Acta.

