# Supplementary material for: Synthesis and Catalytic Properties of Modified Electrodes by Pulsed Electrodeposition of Pt/PANI Nanocomposite
Source: Materials (Basel). 2019 Mar 1;12(5):723. doi: 10.3390/ma12050723 (PMC6427593; doi:10.3390/ma12050723)
Supplement: Supplementary file 1 [file materials-12-00723-s001.pdf]

materials

MDPI

Supporting Materials

Synthesis and Catalytic Properties of Modified Electrodes by Pulsed Electrodeposition of Pt/PANI Nanocomposite

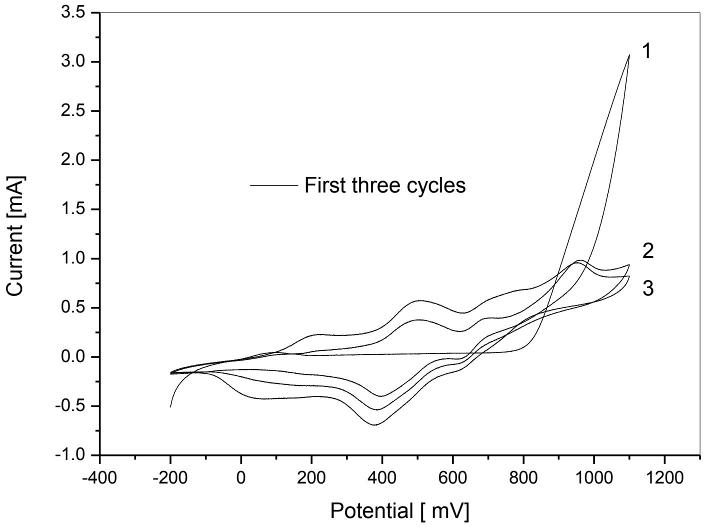

Figure S1. Cyclic voltammetry of aniline polymerisation on a glassy carbon electrode in 0.1 M aniline + 0.5M H_2SO_4 solution at 50 mV/s from −200 to 1100 mV for the first activation cycles.

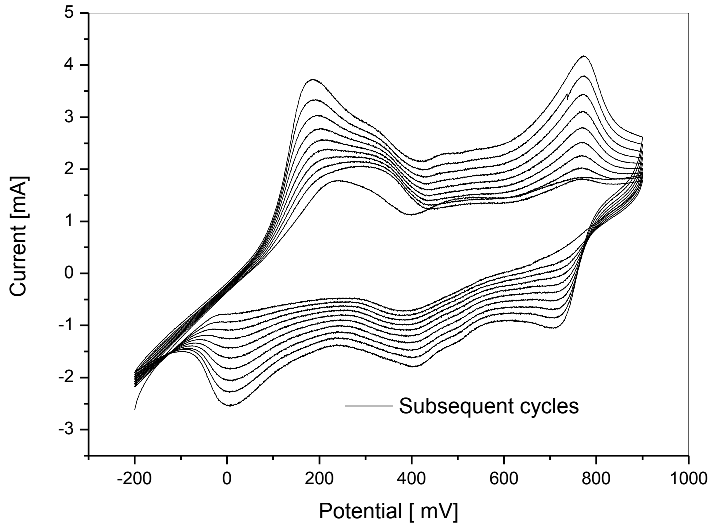

Figure S2. Cyclic voltammetry of aniline polymerization on a glassy carbon electrode in 0.1 M aniline + 0.5 M H_2SO_4 solution at 50 mV/s from −200 to 900 mV for 10 subsequent cycles.

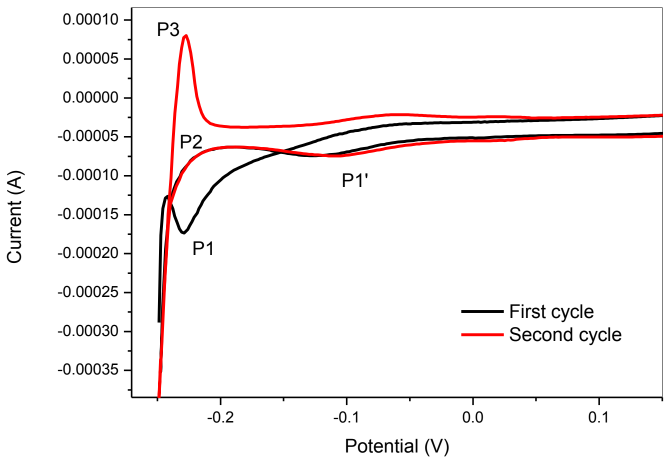

Figure S3. Cyclic voltammetry recorded on glassy carbon electrode at 10 mV/s for 5 mM K_2PtCl_6 + 0.5 M H_2SO_4 plating solution.

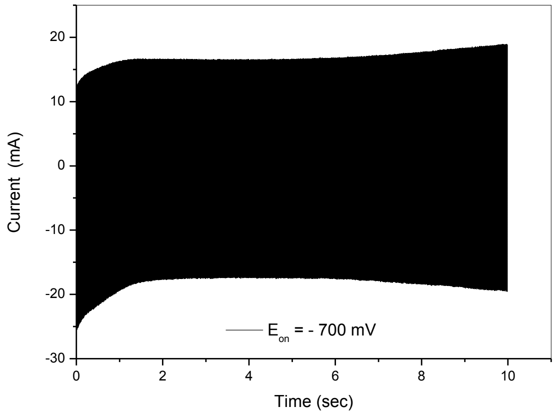

Figure S4. Chronoamperometric curve for potentiostatically electrodeposited platinum on GC/ PANI electrodes at a pulse deposition potential E_{on} = −700 mV. Deposition conditions (0.005 M K_2PtCl_6 in 0.5 M H_2SO_4): t_{on} = 5 ms, E_{off} = +1V, t_{dep} = 5 s, and DC = 50%.

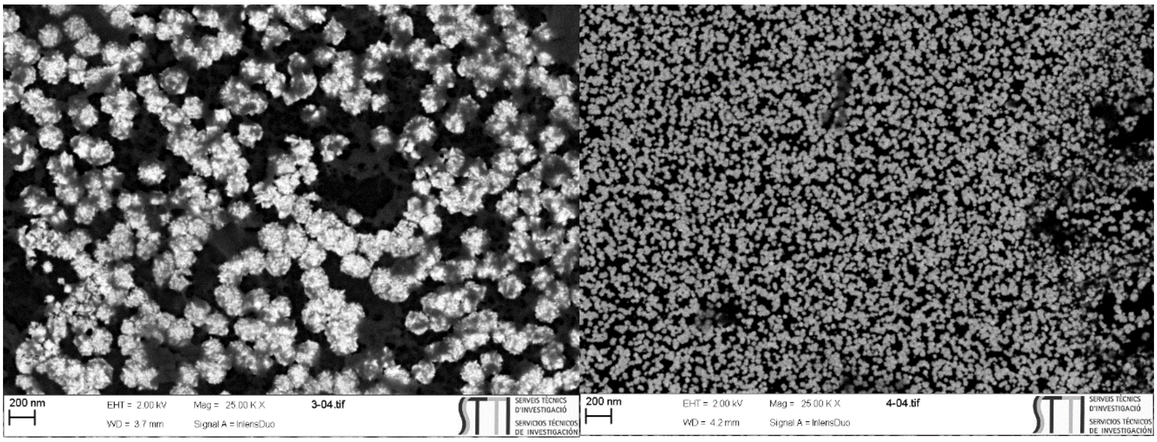

Figure S5. SEM micrographs of GC/PANI(CV)/PtNPs obtained with the following deposition conditions: E_{on} = −500 Mv (left) E_{on} = −750 mV (right), E_{off} = +750 mV, t_{on} = 5 ms, and DC = 50%. 5 mM K_2PtCl_6 in 0.5 M H_2SO_4.
